# Predictors of well child care adherence over time in a cohort of urban Medicaid-eligible infants

**DOI:** 10.1186/1471-2431-11-36

**Published:** 2011-05-15

**Authors:** Anje C Van Berckelaer, Nandita Mitra, Susmita Pati

**Affiliations:** 1 Robert Wood Johnson Foundation Clinical Scholars, University of Pennsylvania, Philadelphia, USA; 2 Leonard Davis Institute of Health Economics, University of Pennsylvania, Philadelphia, USA; 3 Department of Biostatistics and Epidemiology, University of Pennsylvania School of Medicine, Philadelphia, USA; 4 Division of Primary Care Pediatrics, State University of New York at Stony Brook & Stony Brook Long Island Children's Hospital, Stony Brook, USA

## Abstract

**Background:**

Changes in well child care (WCC) adherence over time have not previously been examined. Our objective is to describe adherence rates to WCC over time in a low-income urban population of infants 0-24 months of age, and to identify predictors of WCC adherence in this population.

**Methods:**

This is a secondary analysis of a cohort of Medicaid-eligible children followed from birth to 2 years between 2005 and 2008 with structured telephone surveys to assess maternal well-being, social support, and household and demographic information. For the 260 children attending 4 urban pediatric practices, WCC adherence was assessed based on visit data abstracted from electronic medical records. A random-intercept mixed effects logit model clustered on subject was used.

**Results:**

92% of the mothers were African-American, 27% had not finished high school, 87% were single, and 43% earned < $500/month; mean age was 23. WCC adherence decreased from 88% at 6 months to 47% (12 mo), 44% (18 mo), and 67% (24 mo). The difference across time periods was statistically significant (p < 0.001). Married (OR 1.71, p = 0.02) and primiparous (OR 1.89, p < 0.001) mothers had significantly greater odds of adherence, along with women who reported having been adherent to prenatal care visits (OR 1.49, p = 0.03) and those with the lowest household income (OR 1.40, p = 0.03).

**Conclusions:**

Maternal education efforts should emphasize the importance of establishing WCC, especially for mothers of more than one child. Further studies using larger, more broadly defined populations are needed to confirm our findings that efforts to increase WCC adherence should be intensified after 6 months of age, particularly for children at higher risk.

## Background

Almost 30 million children were covered by Medicaid in 2008 [1]. Though 20% of these children are at significant risk for developmental, behavioral, or social delays [2], only 20-60% receive recommended well child care (WCC), depending on the measure used [2-5]. In high-risk or chronically ill children, adherence to WCC is associated with a lower likelihood of preventable hospitalization [6,7]. Although there is ongoing debate about how WCC should be structured and limited evidence of its efficacy, for children under two years old, the majority of visits coincide with recommended immunizations, for which the evidence is robust [8]. Early and Periodic Screening, Diagnosis, and Treatment (EPSDT) standards, which have formed part of the required Medicaid benefit since 1966, are a cornerstone of pediatric care, mandating the provision of screening and preventive services for children. WCC adherence according to these standards is associated with socioeconomic factors, insurance, and family structure [9], with prenatal care adherence[10,11], with source of care [12] and with maternal risk behavior[4] in some populations. Nonetheless, gaps remain in our understanding of factors within low-income or minority groups that might place children at higher risk of non-adherence. In addition, patterns of adherence over time in this population have not been described.

Much thought has been devoted to the redesign of WCC to better meet family needs and improve outcomes [13-17]. While some interventions have proven successful, their cost precludes a blanket application of these interventions. In order to effectively address the needs of vulnerable children, costly interventions must be effectively targeted to the right children at the right time.

Our objective is to describe adherence rates to WCC over time in a low-income urban population of infants 0-24 months of age, and to identify predictors of WCC adherence in this population. This analysis is part of a larger study examining the influence of child, maternal, and policy-related predictors on continuity of Medicaid coverage among urban medically underserved children.

## Methods

### Study Design and Data Sources

This is a secondary analysis of The Health Insurance Improvement Project, a longitudinal prospective cohort of Medicaid-eligible mothers and their healthy infants designed to determine maternal and child patterns of Medicaid enrollment [18,19]. Between June 15, 2005 and August 6, 2006, 744 study subjects were recruited from the well baby nursery at a large urban hospital shortly after the infant's birth. Inclusion criteria were maternal Medicaid eligibility and maternal English proficiency. Infants who had a gestational age less than 36 weeks, birth weight less than 2500 grams, or who were not in the well baby nursery after delivery were excluded, as were those entering foster care or adoption services. Among eligible dyads, 637 (46%) mothers refused participation and 14 (1%) were missed by the recruiting team, resulting in 744 (53% of eligible infants) enrolled in the study (Additional file 1).

Of this cohort, only those infants for whom ambulatory electronic medical records were available were included (n = 260). Electronic medical records were available from the four urban pediatric practices affiliated with The Children's Hospital of Philadelphia (CHOP) using a single, integrated electronic medical record.

Upon enrollment, mothers completed a baseline survey, which included socio-demographic information, public benefits received and type of health insurance. In addition, each mother was given the short form Test of Functional Health Literacy in Adults (S-TOFHLA). The S-TOFHLA is a well-validated measure of functional health literacy that uses specific health related examples to assess reading comprehension [20]. The short form contains 2 reading passages with scores ranging from 0 to 36 categorized as follows: ≤ 16 limited; 17-22 marginal; ≥ 23 adequate [20].

Subjects were followed for two years from birth with telephone interviews every 6 months assessing child and maternal health and insurance status, household composition, and social support. The Maternal Social Support Index (MSSI) is a validated 21-item questionnaire designed to assess qualitative and quantitative aspects of a mother's social support [21], and was administered at the 12, 18, and 24-month interviews (Table 1).

**Table 1:** Data elements, measurement, and source

Variable	Measure	Data source	Collected
**OUTCOME**			

Adherence to WCC	Categorical: yes or no (from continuous count of visits)	EMR	Once, at end of study

**PREDICTORS**			

• **CHILD**			

Insurance status	2 categories: insured or not	Survey^1^	Every 6 months

Insurance type	5 categories: Medicaid, CHIP, employer-sponsored, individually-purchased, or other	Survey^1^	Every 6 months

Health status	2 categories: fair/poor or good/very good/excellent (dichotomized from 5 categories)	Survey^1^	Every 6 months

• **MOTHER**			

Age	Continuous: years	Survey^1^	Initial

Race	6 categories: White; Black/African American; American Indian/Alaskan Native; Asian; Native Hawaiian/Other Pacific Islander; Some other race	Survey^1^	Initial

Educational status	2 categories: less than high school; high school/GED equivalent/more (dichotomized from 3 categories)	Survey^1^	Initial

Primipara	2 categories: primiparous or not	Survey^1^	Initial

Insurance status	2 categories: insured or not	Survey^1^	Initial & every 6 months

Maternal health literacy	2 categories: adequate or marginal/inadequate (Based on continuous S-TOFHLA score, validated cutpoint at 23 (range 0-36))	Survey	Initial

Receiving any public assistance (WIC, food stamps, SSI)	Categorical: yes or no	Survey	Initial & every 6 months

Maternal health status	Continuous: numerical score from RAND Medical Outcome Study SF-36 subscales	Survey	Initial & every 6 months

Household composition	Categorical: father of baby lives at home or not; other adults live at home or not	Survey^1^	Initial & every 6 months

Maternal employment status	Two categories: full-time employed/student or part-time employed/unemployed looking/unemployed not looking for work Dichotomized from 5 categories	Survey^1^	Initial & every 6 months

Average monthly household income	2 categories: ≤ $500/month or > $500/month Dichotomized at closest value to median from 8 categories: < $250/month; $251 - $500/mo; $501 - $999/mo; $1,000 - $1,499/mo; $1,500 - $1,999/mo; $2,000 - $2,499/mo; $2,500 - $2,999/mo; $3,000 + /mo	Survey^1^	Initial & every 6 months

Maternal social support	Categorical: Maternal Social Support Index (range 0-27) classified as low; medium; or high support by tertiles	Survey	12, 18, and 24 month follow-up

Survey: Data collected in-person or via telephone using survey instrument & questionnaire

^1^Items adapted from the National Health Interview Survey

### Outcome

The primary outcome of interest was complete adherence to the Early Periodic Screening, Diagnosis, and Treatment periodicity schedule for well child care. These visits include one visit by 1 month of age, and visits at 2, 4, 6, 9, 12, 15, 18, and 24 months. By 6 months of age, complete adherence was defined as having had at least 3 well child visits; 2 visits between 6 and 12 months; 2 visits between 12 and 18 months; and one visit between 18 and 24 months (Table 2). For example, for the first six months, a child was coded as adherent if he/she had 3 or more WCC visits in that period, and non-adherent if he/she had fewer. This outcome was abstracted from the electronic medical record: all visits coded as well child visits were counted toward the total in each time period. To validate the content of visits coded as well child care, twenty charts were reviewed (AVB) for inclusion of growth and developmental surveillance as well as review of immunization status and found to be appropriately coded.

**Table 2:** Well child visits included in each time period

*Time period*	*0-< 6 months*	*6-< 12 months*	*12-< 18 months*	*18-< 24 months*
Visits	< 1, 2, 4 months of age (3 visits)	6, 9 months of age (2)	12, 15 months of age (2)	18 months of age (1)
Relaxed criterion (sensitivity analysis)	2 visits	1 visit	1 visit	1 visit

In order to exclude children attending primary care practices outside the network (for whom WCC visits could not be known) from the analysis, for each individual, a 6-month interval with incomplete records was coded as missing. Specifically, incomplete intervals were defined as those with no visits during *and *no visits after that interval, or visits beginning partway through the interval with no preceding visits. Consequently, some subjects have EMR adherence data for only some of the study intervals. Some children transferred care away from and then back into the network; hence, some had data for non-contiguous time periods, such as 0-6 months, and then not again until 18-24 months. In total, 260 of the 744 children were followed for at least one 6-month period in an affiliated practice.

### Predictors

Based on review of the literature about known predictors of health care utilization and adherence[3,9,12,22] as well as clinical relevance, potential predictors included maternal race/ethnicity, maternal health literacy and social support, age, education level, adherence to prenatal care, marital status, employment status, infant birth order, and maternal and child health and health insurance status.

Maternal age and the maternal health subscales from the SF-36 (version 1.0)[23] were kept as the original continuous variables. Among the other independent variables, child health status, maternal educational and employment status, and household income were converted to dichotomous variables from categorical answers. Child health status was grouped into poor/fair and good/very good/excellent to accommodate the small numbers in poor or fair health and to address the potential confounder of interest - that children in poor or fair health would be expected to present more frequently for care, and therefore have more opportunities for preventive care. Maternal educational status was dichotomized with a cutpoint at high school degree; alternative specification with a cutpoint at some college education did not reach significance in the final model. Maternal employment status was dichotomized to reflect full-time activity outside the home, on the one hand, and less than full-time activity (unemployment, part-time employment), on the other. Income, originally collected in 8 categories, was dichotomized using a cut-point of $500/month, which is approximately the median, as the higher income categories included very few subjects. MSSI scores, which were collected as continuous numeric values, were categorized into tertiles for the analysis. The remaining variables were analyzed in their original form.

### Analyses

To determine which family-level independent variables are associated with greater adherence to well child care, we used a logit model for our dichotomous outcome. Specifically, in order to accommodate the structure of the data, which was comprised of a time-varying outcome (adherence at 6, 12, 18, and 24 months) and both fixed (*eg *race) and time-varying covariates (*eg *income, social support), we used a random-intercept mixed effects logit model to assess the relationship between predictors and outcome. This model allowed us to account for the correlation of measurements over time. In our multivariable analysis, covariate inclusion in the model was based on a significance cut-off of p < 0.20 based on univariate analysis.

We estimated the proportion of children adhering to the EPSDT periodicity schedule at each of the four time points and compared them using Cochran's Q for equality of proportions in matched samples [24].

We conducted sensitivity analyses for the definition of our outcome and for missing data. We assessed the sensitivity of our strict adherence outcome definition by relaxing the requirement for being classified as adherent versus non-adherent. Instead of requiring attendance at all recommended visits, we redefined "adherent" to include those who missed at most one visit in each six month window (however, a visit was required in the fourth window because only one visit is recommended in that interval). We also tested our model for sensitivity to the assignment of missing status by reassigning all intervals that include any visit and all intervals following any visit as not missing.

Results are considered to be statistically significant at p < 0.05. All analyses were conducted using STATA, version 10 [25]. This study was approved by the Institutional Review Boards at The Children's Hospital of Philadelphia and the University of Pennsylvania.

## Results

744 mother/baby dyads were enrolled in the study at the time of delivery, and 579 completed at least 6 months of follow-up. 260 of the enrolled children had electronic medical records (EMR) available through their primary care providers affiliated with the Children's Hospital of Philadelphia. 216 of these (83%) remained in the system throughout the full two years. The median number of WCC visits during the two-year observation period in this group was 8 (interquartile range 3). 92% of the subjects for whom EMR are available were African-American. 37% of the mothers were primipara, 87% were unmarried, and 37% earned less than $500 monthly. Those included in the final study sample did not differ significantly from those without electronic records (Table 3). The dyads which did not complete at least 6 months of follow-up did not differ significantly from enrolled dyads in race, education, employment, maternal age, health literacy, country of origin, or birthweight (Additional file 2).

**Table 3:** Characteristics of subjects with and without EMR data

Characteristic	**Have EMR data (final study cohort) (n≈260) **^**§**^		**No EMR data (n≈ 320) **^**§**^		
	**Mean or proportion**	**Range or number**	**Mean or proportion**	**Range or number**	**p**

Maternal age mean	23.2	14-37	23.6	13-45	0.21
Mother's race: African-American	0.92	240	0.92	294	0.95
Marital status: married*	0.12	27	0.12	32	0.77
Maternal education: less than high school	0.27	70	0.34	109	0.06
Monthly household income: < $500*	0.43	97	0.38	98	0.30
Employment status: in school/working full time*	0.46	109	0.48	126	0.64
Mother uninsured*	0.15	36	0.12	31	0.29
Child uninsured*	0.01	3	0.01	3	0.90
Father of baby lives at home*	0.24	57	0.24	57	0.54
Any non-parent adult lives at home*	0.37	87	0.34	88	0.47
Primipara	0.38	97	0.34	107	0.31
Maternal social support index* mean	21.1	6-36	21.0	6-36	0.58
1^st ^tertile (proportion)	0.36	74	0.37	87	0.97
2^nd ^tertile	0.34	70	0.33	79	
3^rd ^tertile	0.31	64	0.30	71	
Maternal S-TOFHLA score mean**	29.5	2-36	28.3	2-36	0.95
Child health status: poor or fair*	0.03	6	0.05	14	0.11
Adherence to prenatal care: all of the time (maternal report)	0.78	204	0.73	232	0.13

^§^Denominator varies as not every answer had 100% response.

*time-varying covariates: proportions and means reflect the interview at 12 months of age

**short form of the Test of Functional Health Literacy: "adequate" score is ≥23

**Figure 1:**
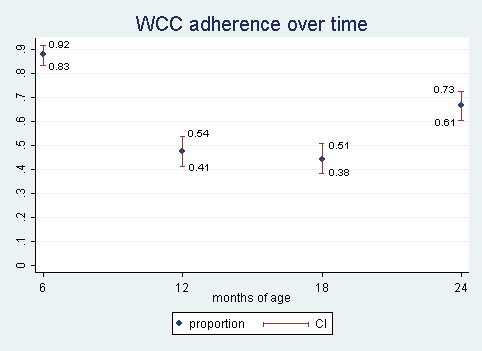
**Well child care adherence by age**.

Among children for whom EMR data were available, WCC adherence changed from 88% at 6 months to 47% (12 mo), 44% (18 mo), and 67% (24 mo). The difference across time periods was statistically significant (p < 0.001) (Figure 1). The mean (SD) number of WCC visits in the first six months was 3.68 (1.07); for 6-11 months 1.35 (0.85); for 12-17 months 1.39 (0.76); and for 18-24 months 0.69 (0.51).

In unadjusted mixed-effects regression analysis, we found that specific family sociodemographic characteristics and prior health care utilization were associated with increased likelihood of WCC adherence. Specifically, mothers whose household income was less than $500 per month had 1.42 times greater odds of adhering to WCC than mothers with incomes over $500 per month. Mothers who had only one child had 1.77 times greater odds of adhering to the WCC schedule, compared to mothers of more than one child. Moreover, mothers who reported receiving all recommended prenatal care had 1.80 times greater odds of adherence than mothers who did not. In addition to these predictors, maternal health insurance status, marital status, and presence of another non-parent adult in the home all met our *a priori *criterion (p < 0.2) for inclusion in the multivariable model (Table 4).

**Table 4:** Unadjusted odds ratios from mixed-effects regression models for characteristics associated with WCC adherence

	OR (95% CI)
Mother < 20 years of age (reference: ≥20 years of age)	1.16 (0.84-1.58)
African-American (reference: all other races)	0.80 (0.47-1.36)
Mother married* (reference: all other marital statuses)	1.34 (0.88-2.05)
Mother graduated high school (reference: did not graduate high school)	1.15 (0.84-1.57)
Income < $500/mo* (reference: ≥$500/month)	1.42 (1.01-1.90)
Mother is student or employed full-time (reference: unemployed, employed part-time)	0.93 (0.70-1.23)
Mother insured * (reference: not insured)	1.31 (0.90-1.90)
Child insured (reference: not insured)	1.73 (0.70-4.31)
Father of baby lives at home (reference: does not live at home)	0.92 (0.66-1.28)
Any nonparent adult at home* (reference: no non-parent adults at home)	1.30 (0.97-1.75)
Primipara* (reference: multipara)	1.77 (1.34-2.34)
MSSI (tertiles)	1.04 (0.84-1.29)
Adequate TOFHLA-S literacy score (≥23) (reference: < 23)	0.95 (0.69-1.32)
Child in poor/fair health (reference: good/excellent health)	0.72 (0.36-1.45)
Mother's SF-36 subscales (reference: a one-point difference in the score)	
Physical function	1.00 (1.00-1.01)
Physical limitation	1.00 (1.00-1.01)
Emotional limitation	1.00 (0.99-1.00)
Energy	1.00(0.99-1.00)
General health	1.00(0.99-1.01)
Emotional wellness	1.00(0.99-1.00)
Maternal report of PNC adherence* (reference: maternal report of PNC adherence less than 'all of the time')	1.80 (1.30-2.50)
Receiving any public assistance (WIC, food stamps, SSI) (reference: not receiving any public assistance)	0.93 (0.55-1.59)

*p < 0.20--meets *a priori *criterion for inclusion in the model

After multivariable adjustment, our findings about the influence of some maternal individual sociodemographic characteristics and prior health care utilization patterns remained unchanged. Specifically, married (OR 1.71, 95% CI: 1.09-2.69) and primiparous (OR 1.87, 95% CI: 1.36-2.63) mothers had significantly greater odds of adherence than single mothers and mothers with more than one child, respectively. We tested for interaction between marital status and parity, and found no significant effect. Mothers in the lowest income category also remained significantly more likely to adhere to WCC visits (OR 1.40, 95% CI 1.03-1.91). In addition, women who reported having been adherent to prenatal care visits had 1.56 times (95% CI: 1.09-2.23) greater odds of adherence than mothers who did not adhere to recommended prenatal care visits. In contrast, after multivariable adjustment, presence of any other adult in the household and maternal insurance did not significantly affect adherence (Table 5).

**Table 5:** Multivariable mixed-effects regression analysis of WCC adherence

*Predictor*	*OR (95% CI)*	*p*
Primipara	1.89 (1.36-2.63)	< 0.001
Mother insured	1.31 (0.89-1.92)	0.17
Married mother	1.71 (1.09-2.69)	0.02
Other non-parent adult at home (reference: no non-parent adults at home)	0.98 (0.71-1.35)	0.89
Income < $500/month (reference group: ≥$500/month)	1.40 (1.03-1.91)	0.03
Adherent to prenatal care	1.49 (1.05-2.12)	0.03

Because of the concern about endogeneity when including reported prenatal care adherence as a predictor, we tested the model for sensitivity by excluding this predictor, and did not find significant changes in the odds for other predictors. We validated our definition of adherence for the outcome by comparing it to an alternative, more relaxed definition. With this alternative coding, less than half as many observed six-month periods were coded as non-adherent (164 vs. 392). In the model using this outcome coding, the odds ratios maintained their direction of effect, but only income and prenatal care adherence remained significant. Odds ratios are shown in additional file 3. We also tested our model for sensitivity to the assignment of missing intervals. This resulted in an additional 130 observed intervals distributed across the four six-month time periods. 89% of the newly included intervals met our criterion for nonadherence. Using this expanded dataset in the model, parity and prenatal care remained significant, while marital status and income retained the direction of their effect, but were no longer significant (Additional file 3).

Because income level was missing in a relatively large portion of women (10-29% of observations, varying by interview period), we assessed the robustness of our results by conducting two sensitivity analyses: in the first analysis, we assigned all missing income levels a value of ≥$500/month and in the second analysis, we assigned all missing incomes to the < $500/month category. Our findings are robust to these two extremes: when all missing observations were assigned to either the higher or lower income, all of our previously significant predictors except income remained statistically significant and the effect estimates (odds ratios) were minimally affected. Previously non-significant predictors such as insurance and other adults in the home remained non-significant (Table 6).

**Table 6:** Sensitivity tests for missing income values: multivariable mixed effects regression analysis of WCC adherence

	missing income assigned lower value			missing income assigned higher value		
Predictor	OR	CI	p	OR	CI	p
Primipara	1.85	1.34-2.55	< 0.001	1.90	1.39-2.62	< 0.001
Mother insured	1.30	0.89-1.90	0.16	1.32	0.91-1.92	0.14
Married mother	1.71	1.10-2.66	0.02	1.71	1.10-2.67	0.02
Other adult at home	1.03	0.75-1.41	0.86	1.05	0.76-1.43	0.78
Income < $500/month	1.35	1.00-1.81	0.05	1.28	0.95-1.73	0.10
Adherent to prenatal care	1.46	1.04-2.05	0.03	1.44	1.02-2.02	0.04

## Discussion

Our principal finding is that adherence to WCC schedules decreases after 6 months of age in this low-income urban population. This drop in adherence in a largely publicly insured population (> 90% at each time point) has not previously been described, and reveals a missed opportunity in preventive health services for these high-risk children. Why this drop should occur specifically after 6 months is not clear from our data. Other studies have found that parents and providers have different expectations of well child visits, and that parents' expectations are poorly met [13]. We hypothesize that care for younger infants is perceived as a higher priority, either because of greater parental uncertainty or greater perceived vulnerability of the infant. Other researchers have shown a trend toward greater utilization of preventive services under 1 year of age, as compared to after 1 year [9,12], but did not address this trend specifically. Of concern, the great majority of well child visits in the age range we studied are associated with immunizations. In a population already at higher risk of under-immunization[26] and unrecognized developmental delays[27], missed visits likely contribute to these problems. The rise in adherence in the 18-< 24 month age group may be due to the front-loaded structure of the EPSDT schedule: with only one recommended visit in that time period, it is easier to adhere to the schedule in that time period than 6-< 12 or 12-< 18 months, when 2 visits are expected in each.

In addition to the subjects' changing adherence trajectory, we found that adherence was predicted most strongly by maternal prenatal care adherence, number of other children, maternal marital status, and low income. Prenatal care adherence and birth order as predictors of adherence are consistent with previous findings [26,28]. Birth order also influences early immunization status in this same cohort [19]. We hypothesize that single mothers and mothers of more than one child may be less able to attend preventive visits due to competing needs of other children. An alternative but not exclusive explanation is that mothers of more than one child feel more confident in the care of their younger infants. In either case, mothers must feel there is value in a health service if they are to prioritize it among the many other needs of their children.

Sensitivity analyses testing the robustness of our findings against alternative specifications of the outcome and missing data maintained significant associations between parity, prenatal care adherence, and income, and adherence, depending on the specification. In contrast, while marital status maintained the direction of its effect, it was no longer significantly associated with the outcome. Further study is needed to clarify this relationship.

The finding that mothers in the lowest income bracket are more likely to adhere to WCC may be associated with the fact that these families are more likely to be eligible for other kinds of services. Indeed, mothers in this category were themselves more likely to have health insurance as the income threshold for insurance coverage in adults is considerably lower than for young children.

There are some limitations to this study. First, our study population was comprised primarily of African Americans (92%) from the Philadelphia metropolitan area. Hence, our results have limited generalizability to other races/ethnicities or to suburban or rural settings. While non-English speakers were excluded, they represent a small proportion of the Medicaid-eligible population in the hospital from which patients were recruited. The number of individuals lost to follow-up may have also introduced additional bias in the sample, however, comparisons between retained subjects and those lost do not reveal significant differences between them. Second, while we attempted to include only those subjects and time periods in which children received care principally within the EMR system, it is possible that some subjects may have attended well child visits outside the system to which we had access. Consequently, the rates of adherence we report may be an underestimate. However, our results are consistent with published adherence rates for similar populations [3-5]. Because the majority of Medicaid enrollees in the Philadelphia region are enrolled in managed care organizations which require an assigned primary care provider, patients should see that provider for all their primary care needs within a given period, which mitigates this underestimation. We restricted outcome data to those children in whom we could be reasonably confident that they had been consistently assigned to the practice from which we gathered the EMR data. Third, because this is a secondary analysis of a dataset not specifically designed to assess well child care adherence, the survey did not include maternal report of well child visits, which would obviate the need for abstracting data from the medical record and excluding subjects for whom records were not available, yielding a larger sample size.

Finally, as with any survey, there is potential for biased recall or social desirability bias. Among the predictors, the most likely to be sensitive to this would be income. Indeed, this was the variable with the greatest number of missing observations; however, our sensitivity analyses showed that our findings were robust to these missing data. Despite these limitations, this study furthers our understanding of predictors of WCC adherence in young children.

## Conclusions

The drop in WCC adherence by up to 50% after 6 months of age represents both an opportunity for intervention and an avenue for further investigation. Additional studies of adherence over time in more diverse groups are needed. Maternal education efforts should emphasize the importance of establishing WCC, especially for mothers of more than one child. Several interventions have attempted to improve the delivery of WCC [17,29,30]. Our findings suggest that, in low-income urban African-American children, these efforts should not be restricted to early infancy, but should be intensified after 6 months of age, particularly for children at higher risk.

## Competing interests

The authors declare that they have no competing interests.

## Authors' contributions

AVB conceived of the study, performed the statistical analysis, and drafted the manuscript. NM participated in the design of the study and revision of the manuscript. SP participated in the design of the study and revision of the manuscript, and conceived of the original study that led to development of the cohort. All authors read and approved the final manuscript.

## Pre-publication history

The pre-publication history for this paper can be accessed here:

http://www.biomedcentral.com/1471-2431/11/36/prepub

## Supplementary Material

Additional file 1 **Study enrollment protocol**. Click here for file

Additional file 2 **Population characteristics for all enrollees vs. those lost to follow-up**. Click here for file

Additional file 3 **Sensitivity analysis for outcome - relaxed adherence criterion and missing intervals**. Click here for file

## References

[B1] Annual Statistical Enrollment Report FY2008 http://www.cms.hhs.gov

[B2] Bethell C Peck C Abrams M Halfon N Sareen H Collins K Partnering With Parents to Promote the Healthy Development of Young Children Enrolled in Medicaid 2002 New York, NY: The Commonwealth Fund

[B3] Houtrow AJ Kim SE Chen AY Newacheck PW Preventive health care for children with and without special health care needs Pediatrics 2007 119 4 e821 828 10.1542/peds.2006-1896 17403825 PMC2367154

[B4] Hambidge SJ Phibbs SL Chandramouli V Fairclough D Steiner JF A stepped intervention increases well-child care and immunization rates in a disadvantaged population Pediatrics 2009 124 2 455 464 10.1542/peds.2008-0446 19651574

[B5] Gavin NI Adams EK Herz EJ Chawla AJ Ellwood MR Hill IT Zimmerman BL Wasserman J The use of EPSDT and other health care services by children enrolled in Medicaid: the impact of OBRA'89 Milbank Q 1998 76 2 207 250 10.1111/1468-0009.00087 9614421 PMC2751077

[B6] Hakim RB Bye BV Effectiveness of compliance with pediatric preventive care guidelines among Medicaid beneficiaries Pediatrics 2001 108 1 90 97 10.1542/peds.108.1.90 11433059

[B7] Tom JO Tseng CW Davis J Solomon C Zhou C Mangione-Smith R Missed well-child care visits, low continuity of care, and risk of ambulatory care-sensitive hospitalizations in young children Arch Pediatr Adolesc Med 2010 164 11 1052 1058 10.1001/archpediatrics.2010.201 21041598 PMC3551592

[B8] Committee on Infectious Diseases Recommended Childhood and Adolescent Immunization Schedules--United States, 2010 Pediatrics 2010 125 1 195 196 20048088 10.1542/peds.2009-3194

[B9] Selden TM Compliance with well-child visit recommendations: evidence from the Medical Expenditure Panel Survey, 2000-2002 Pediatrics 2006 118 6 e1766 1778 10.1542/peds.2006-0286 17142499

[B10] Butz AM Funkhouser A Caleb L Rosenstein BJ Infant health care utilization predicted by pattern of prenatal care Pediatrics 1993 92 1 50 54 8516084

[B11] Kogan MD Alexander GR Jack BW Allen MC The association between adequacy of prenatal care utilization and subsequent pediatric care utilization in the United States Pediatrics 1998 102 1 Pt 1 25 30 9651409 10.1542/peds.102.1.25

[B12] Freed GL Clark SJ Pathman DE Schectman R Influences on the receipt of well-child visits in the first two years of life Pediatrics 1999 103 4 Pt 2 864 869 10103323

[B13] Schor EL Rethinking well-child care Pediatrics 2004 114 1 210 216 10.1542/peds.114.1.210 15231930

[B14] Radecki L Olson LM Frintner MP Tanner JL Stein MT What Do Families Want From Well-Child Care? Including Parents in the Rethinking Discussion Pediatrics 2009 124 3 858 65 10.1542/peds.2008-2352 19706562

[B15] Coker TR Chung PJ Cowgill BO Chen L Rodriguez MA Low-income parents' views on the redesign of well-child care Pediatrics 2009 124 1 194 204 10.1542/peds.2008-2608 19564300 PMC4587564

[B16] Schuster MA Wood DL Duan N Mazel RM Sherbourne CD Halfon N Utilization of well-child care services for African-American infants in a low-income community: results of a randomized, controlled case management/home visitation intervention Pediatrics 1998 101 6 999 1005 10.1542/peds.101.6.999 9606226

[B17] Margolis PA Stevens R Bordley WC Stuart J Harlan C Keyes-Elstein L Wisseh S From concept to application: the impact of a community-wide intervention to improve the delivery of preventive services to children Pediatrics 2001 108 3 E42 10.1542/peds.108.3.e42 11533360

[B18] Pati S Mohamad Z Cnaan A Kavanagh J Shea JA Influence of maternal health literacy on child participation in social welfare programs: the Philadelphia experience Am J Public Health 2010 100 9 1662 1665 10.2105/AJPH.2009.172742 20634468 PMC2920956

[B19] Pati S Feemster KA Mohamad Z Fiks A Grundmeier R Cnaan A Maternal Health Literacy and Late Initiation of Immunizations Among an Inner-City Birth Cohort Matern Child Health J 2011 15 3 386 94 10.1007/s10995-010-0580-0 20180003 PMC6733024

[B20] Nurss J Parker R Williams M Baker D Test of Functional Literacy in Adults 2001 2 Snow Camp, NC: Peppercorn Books & Press

[B21] Pascoe JM Ialongo NS Horn WF Reinhart MA Perradatto D The reliability and validity of the maternal social support index Fam Med 1988 20 4 271 276 3203834

[B22] Selden TM Hudson JL Access to care and utilization among children: estimating the effects of public and private coverage Med Care 2006 44 5 Suppl I19 26 16625060 10.1097/01.mlr.0000208137.46917.3b

[B23] Stewart A Sherbourne C Hays R Stewart A, Ware J Summary and discussion of MOS measures Measuring functioning and well-being: the medical outcome study approach 1992 Durham, NC: Duke University Press 345 371

[B24] Cochran WG The comparison of percentages in matched samples Biometrika 1950 37 256 266 14801052

[B25] StataCorp Stata Statistical Software: Release 10 2007 College Station, TX: StataCorp LP

[B26] Wood D Donald-Sherbourne C Halfon N Tucker MB Ortiz V Hamlin JS Duan N Mazel RM Grabowsky M Brunell P Freeman H Factors related to immunization status among inner-city Latino and African-American preschoolers Pediatrics 1995 96 2 Pt 1 295 301 7630688

[B27] Wertheimer R Croan T Moore KA Hair EC Attending Kindergarten and Already Behind: A Statistical Portrait of Vulnerable Young Children Child Trends 2003 Washington, DC

[B28] Bardenheier BH Yusuf HR Rosenthal J Santoli JM Shefer AM Rickert DL Chu SY Factors associated with underimmunization at 3 months of age in four medically underserved areas Public Health Rep 2004 119 5 479 485 10.1016/j.phr.2004.07.005 15313111 PMC1497657

[B29] Bergman DA Beck A Rahm AK The use of internet-based technology to tailor well-child care encounters Pediatrics 2009 124 1 e37 43 10.1542/peds.2008-3385 19564267

[B30] Szilagyi PG Schaffer S Shone L Barth R Humiston SG Sandler M Rodewald LE Reducing geographic, racial, and ethnic disparities in childhood immunization rates by using reminder/recall interventions in urban primary care practices Pediatrics 2002 110 5 e58 10.1542/peds.110.5.e58 12415064

