# Supplementary material for: Predictors of well child care adherence over time in a cohort of urban Medicaid-eligible infants
Source: BMC Pediatr. 2011 May 15;11:36. doi: 10.1186/1471-2431-11-36 (PMC3118120; doi:10.1186/1471-2431-11-36)
Supplement: Additional file 1 — Study enrollment protocol. [file 1471-2431-11-36-S1.DOC]

**Additional file 1. Study enrollment protocol**

3451 live births between 6/15/05-8/6/06

2680 infants met inclusion criteria to be screened (birthweight>2500 grams and gestational age >36 weeks)

1395 mother-infant dyads eligible for study

744 mother-infant dyads agree to participate

1147 not Medicaid-eligible or not

living in Pennsylvania

103 not English proficient

35 not in maternal custody

637 refuse to participate

14 missed by recruiters

260 records with electronic medical record data

580 completed the 6 month survey

320 received primary care entirely at outside facilities

164 did not complete 6 month survey
