# Supplementary material for: Predictors of well child care adherence over time in a cohort of urban Medicaid-eligible infants
Source: BMC Pediatr. 2011 May 15;11:36. doi: 10.1186/1471-2431-11-36 (PMC3118120; doi:10.1186/1471-2431-11-36)
Supplement: Additional file 2 — Population characteristics for all enrollees vs. those lost to follow-up. [file 1471-2431-11-36-S2.DOC]

**Additional file 2.** Population characteristics for all enrollees vs. those lost to follow-up

|  | ***No Six Month Data (N=164)*** | ***Enrolled***  ***(N=744)*** | **p-value** |
| --- | --- | --- | --- |
| **Race (%)** |  |  | 0.07 |
| Black | 132 (80) | 604 (81) |  |
| Other | 32 (20) | 140 (19) |  |
| **Education (%)** |  |  | 0.15 |
| Less than high school | 63 (34) | 243 (33) |  |
| High school | 44 (27) | 182 (24) |  |
| More than high school | 57 (35) | 319 (43) |  |
| **Employment (%)** |  |  | 0.89 |
| Student | 46 (28) | 233 (31) |  |
| Employed | 50 (31) | 224 (30) |  |
| Unemployed (looking for work) | 20 (12) | 78 (11) |  |
| Unemployed (no looking for work) | 21 (13) | 84 (11) |  |
| Missing | 27 (16) | 125 (17) |  |
| **Maternal Age (%)** |  |  | 0.93 |
| > 20 | 36 (22) | 184 (25) |  |
| 20 - 24 | 67 (41) | 300 (40) |  |
| 25 - 29 | 37 (23) | 159 (21) |  |
| 30 - 34 | 16 (10) | 72 (10) |  |
| > 34 | 8 (5) | 29 (4) |  |
| **Maternal Health Literacy^1^** |  |  | 0.14 |
| Inadequate/Marginal | 26 (17) | 158 (22) |  |
| Adequate | 129 (83) | 558 (78) |  |
| **Country (%)^2^** |  |  | 0.13 |
| US born | 145 (88) | 685 (92) |  |
| Non-US born | 19 (12) | 59 (8) |  |
| **Baby Weight (IQR)** | 3249 (2943 – 3490) | 3282 (2970 – 3553) | 0.06 |
| **Maternal Age (IQR)** | 24 (20 – 26) | 23 (20 – 26) | 0.45 |

^1^Only 716 from the enrolled category had a S-TOFHLA score because this was administered only to those who enrolled after July 15, 2005.

^2^There are 22 with missing data for this item.
