# Supplementary material for: Predictors of well child care adherence over time in a cohort of urban Medicaid-eligible infants
Source: BMC Pediatr. 2011 May 15;11:36. doi: 10.1186/1471-2431-11-36 (PMC3118120; doi:10.1186/1471-2431-11-36)
Supplement: Additional file 3 — Sensitivity analysis for outcome - relaxed adherence criterion and missing intervals. [file 1471-2431-11-36-S3.DOC]

Additional file 3:

**Sensitivity analysis for outcome – relaxed adherence criterion and missing intervals**

|  | ***Relaxed adherence criterion*** | | ***Reassignment of partial intervals as non-missing*** | |
| --- | --- | --- | --- | --- |
| ***Predictor*** | ***OR (95% CI)*** | ***p*** | ***OR (95% CI)*** | ***p*** |
| Primipara | 1.57 (1.00-2.50) | 0.05 | 1.59 (1.13-2.23) | <0.007 |
|  |
| Mother insured | 1.22 (0.74- 2.01) | 0.44 | 1.07 (0.72-1.58) | 0.75 |
| Married mother | 1.14 (0.62-2.08) | 0.67 | 1.55 (0.95-2.52) | 0.08 |
| Other adult at home | 0.75 (0.49-1.16) | 0.20 | 1.04 (0.76-1.44) | 0.80 |
| Income < $500/month | 2.01 (1.29-3.14) | 0.002 | 1.31 (0.97-1.78) | 0.08 |
| Adherent to prenatal care | 1.67 (1.07-2.60) | 0.02 | 1.57 (1.08-2.28) | 0.02 |
